# Traumatic Dental Injuries Among Individuals with Disabilities and Chronic Diseases Practicing Sports

**DOI:** 10.3390/jcm14144995

**Published:** 2025-07-15

**Authors:** Karolina Gerreth, Alicja Hoffmann-Przybylska, Marianna Kicerman, Mark Alejski, Piotr Przybylski

**Affiliations:** Department of Risk Group Dentistry, Poznan University of Medical Sciences, 70 Bukowska Street, 60-812 Poznan, Poland; alicjahoffmann21@gmail.com (A.H.-P.); mkicerman@ump.edu.pl (M.K.); malejski@ump.edu.pl (M.A.); piotrprzybylski@ump.edu.pl (P.P.)

**Keywords:** dental traumatic injuries, special needs athletes, intellectual disability, chronic diseases

## Abstract

**Background/Objectives:** Participation in sports activities is one of the risk factors for traumatic dental injuries. Nevertheless, little data are available in the literature on such problems in persons with disabilities. This study aims to evaluate the prevalence and severity of traumatic dental injuries in athletes with intellectual disabilities and other coexisting chronic diseases, as well as the use of mouthguards and the level of treatment of injuries in this population. **Methods:** The research was carried out in seven special needs schools. Two calibrated dentists performed dental examinations in 100 subjects practicing sports, aged 8–30 years (study group), and in 128 individuals, aged 8–25 years, who do not perform systematic physical activity (control group). Statistica Software v.10 was used for statistical analysis, with the level of statistical significance at *p* ≤ 0.05. **Results:** The majority of individuals had one tooth affected by traumatic injury in the study and control individuals, with the results amounting to 14% and 5%, respectively; the difference between both groups was statistically significant (*p* = 0.02). Only one athlete used a mouthguard during training and competitions. Restorative treatment of traumatically damaged teeth was performed in six athletes (37%) out of the total sixteen subjects affected by dental injuries from the study group and in two (15%) out of thirteen participants from the controls. **Conclusions:** This study reveals that dentists should be professionally prepared to meet the special needs of the population with disabilities and chronic disorders to minimize the burden of dental trauma. There is an urgent need for preventive programs for special needs athletes, their parents/caregivers, and trainers concerning the use of mouthguards.

## 1. Introduction

The systematic practice of sport or any other physical activity provides people of different ages and conditions, including individuals with disabilities and chronic illnesses, with a wide range of mental, social, and physical health benefits [1]. A lack of physical activity in persons with an intellectual disability may be linked with numerous disorders, including heart and muscle diseases, obesity, and type II diabetes [1,2,3].

Literature data emphasize the positive effects of physical activity on increasing functional mobility, power, agility, speed, reaction time, and physical fitness [4,5]. Moreover, it can enhance quality of life and perception of well-being, as well as possibly having a positive effect on self-esteem, social competence, attitude, behaviour, self-concept, and cardiovascular endurance [4,6]. Furthermore, cardiorespiratory fitness is also definitely related to academic performance and mental health in children [7]. People playing a sport of their choice are usually intrinsically motivated to participate in such an activity. This is often associated with self-efficacy, enjoyment, and recreational rather than competitive play [6].

Promotion of physical activity in children and adolescents is vital since a physically active lifestyle, as well as other behaviours such as toothbrushing, start to develop very early in childhood [1]. It is emphasized that preschool age is a proper and essential time for physical fitness development since, on the one hand, it promotes normal growth, and on the other, exerts carryover effects that positively affect quality of life and personal health later in adulthood [7].

However, participation in different sports activities is one of the major risk factors for traumatic dental and oral injuries, especially in basketball, soccer (association football), and boxing [8]. The vast majority of traumatic dental injuries involve the maxillary anterior teeth [9]. Moreover, predisposing factors for dental trauma could be related to the individual’s anatomic features, such as inadequate lip coverage of the upper anterior teeth or increased overjet [9]. However, in individuals with an intellectual disability and/or other general diseases, traumatic injuries are also related to other factors [10]. Interestingly, cerebral palsy is considered the major reason for motor impairment in young children, and the majority of sufferers live with coexisting impairments [11], such as epilepsy [12]. Therefore, injuries of the teeth or other orofacial structures could also be observed during falls associated with a loss of balance or epileptic seizures [12,13,14].

Scarce data available from the literature indicate that the most common form of traumatic dental injury in athletes with an intellectual disability is a fracture that involves only the enamel of the tooth (Ellis class 1 fractures), and subsequently a fracture that involves enamel and dentin excluding pulp (Ellis class 2 fractures). Most of the trauma involves the maxillary incisors [8].

It must be highlighted that traumatic dental injuries could have a significant impact not only on the sufferers and their families, but also on dental healthcare professionals [15,16]. Treatment of traumatic dental injuries in patients with disabilities is not an ordinary situation in daily dental practice. Nevertheless, this issue remains underrepresented within research concerning paediatric oral health [15]. Moreover, studies investigating the social determinants of dental trauma are still scarce, but are nevertheless greatly required to inform health promotion strategies and help in the prevention of its occurrence [16].

Needless to say, traumatic dental injuries in sports may be quite easily prevented, or their severity and number might be decreased due to the use of mouthguards [17]. The use of this device in contact sports is strongly recommended, as it can absorb the impact of force, thereby protecting the teeth.

To the best of our knowledge, currently, little is known about the prevalence of dental trauma among individuals with intellectual disabilities, including those participating in different sports activities. Moreover, most studies do not include detailed analysis, i.e., concerning the prevalence and/or severity of traumatic damage to the teeth, the type of injury, and a wide range of characteristics of two groups of patients with intellectual disabilities (one practicing sports and the other undertaking no physical activity).

Therefore, the present study aims to assess the prevalence and severity of orofacial injuries in athletes with an intellectual disability and other coexisting general diseases, as well as the use of mouthguards and the level of treatment of dental trauma in this population.

The null hypothesis of this study is that differences are present in the prevalence of orofacial injuries in athletes with an intellectual disability and in individuals from the control group who do not practice sports.

## 2. Materials and Methods

### 2.1. Study Population

The research included athletes with intellectual disabilities (study group) who physical education teachers coached in special needs schools. Other students from the same educational institutions were analysed as a control group.

Prior to the study, each parent/legal caregiver was provided with a pamphlet, which was delivered to him/her by the coach (study group) or the class teacher during the parent–teacher meeting, detailing the examination. Each parent/caregiver also received a form of informed consent as well as a sociomedical survey about their child’s traumatic injuries. Afterwards, the forms with written and informed consent provided by the parents/caregivers and completed sociomedical surveys were delivered to the examiners by the trainers and teachers. Full confidentiality of the gathered information was ensured for all participants of this study.

The examined individuals in both the study and control groups had intellectual disabilities, often coexisting with other systemic disorders and/or additional disabilities (physical and/or sensory).

The athletes (children, youths, and young adults) that participated in the dental examination were not randomly recruited by the dentists; all special needs students who actively practice sports at the school and those who take part in Special Olympics across six schools in Poznan and one school in Szamotuly (Wielopolska province, western Poland) were invited and constituted the study group. In accordance with the trainers’ suggestion, the researchers decided to examine athletes who are also Special Olympics participants at the schools to allow for a longer examination time for a single participant and to ensure that every athlete was examined only once.

Each patient’s chart was much more detailed than the one used during the Special Olympics Special Smile oral assessment [18]. The control group consisted of all other special needs students who attended the same schools.

**Figure 1:**
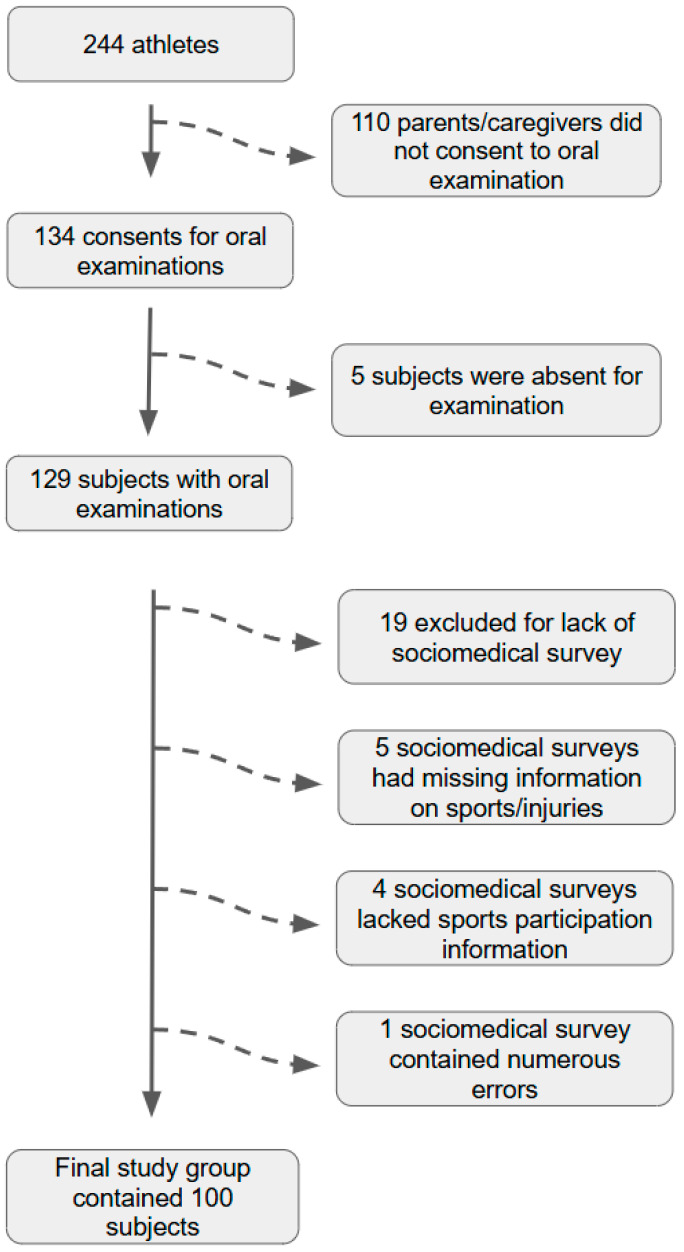
A flow diagram explaining how the final study group was selected based on the initial group of athletes who met all of the inclusion criteria.

Before the study, it was confirmed that 244 athletes attended the aforementioned establishment/schools, and all their parents/caregivers received a pamphlet concerning the research, a consent form for dental examination, and a sociomedical survey (Figure 1). After two weeks, 134 consent forms and 115 completed sociomedical surveys were collected. The examiners visited each school 1–3 times to carry out dental examinations, since there were too many subjects and some of the students were absent during the days on which examinations were performed. Finally, 129 children had dental examinations performed (5 individuals were absent during each visit). In the analysis, only the sets of properly completed sociomedical surveys and patients’ charts were taken into account. Therefore, 19 subjects who underwent an oral examination but whose parents/caregivers did not return the sociomedical survey, 5 individuals whose parents completed the sociomedical survey but they did not undergo an oral examination since they were absent from school during the days of examination or they were uncooperative, 5 students whose parents/caregivers did not answer the questions concerning playing sports as well as injuries, 4 subjects whose sociomedical surveys lacked information on sports participation, and 1 athlete whose parents made numerous mistakes in the sociomedical survey were excluded from further analysis.

**Figure 2:**
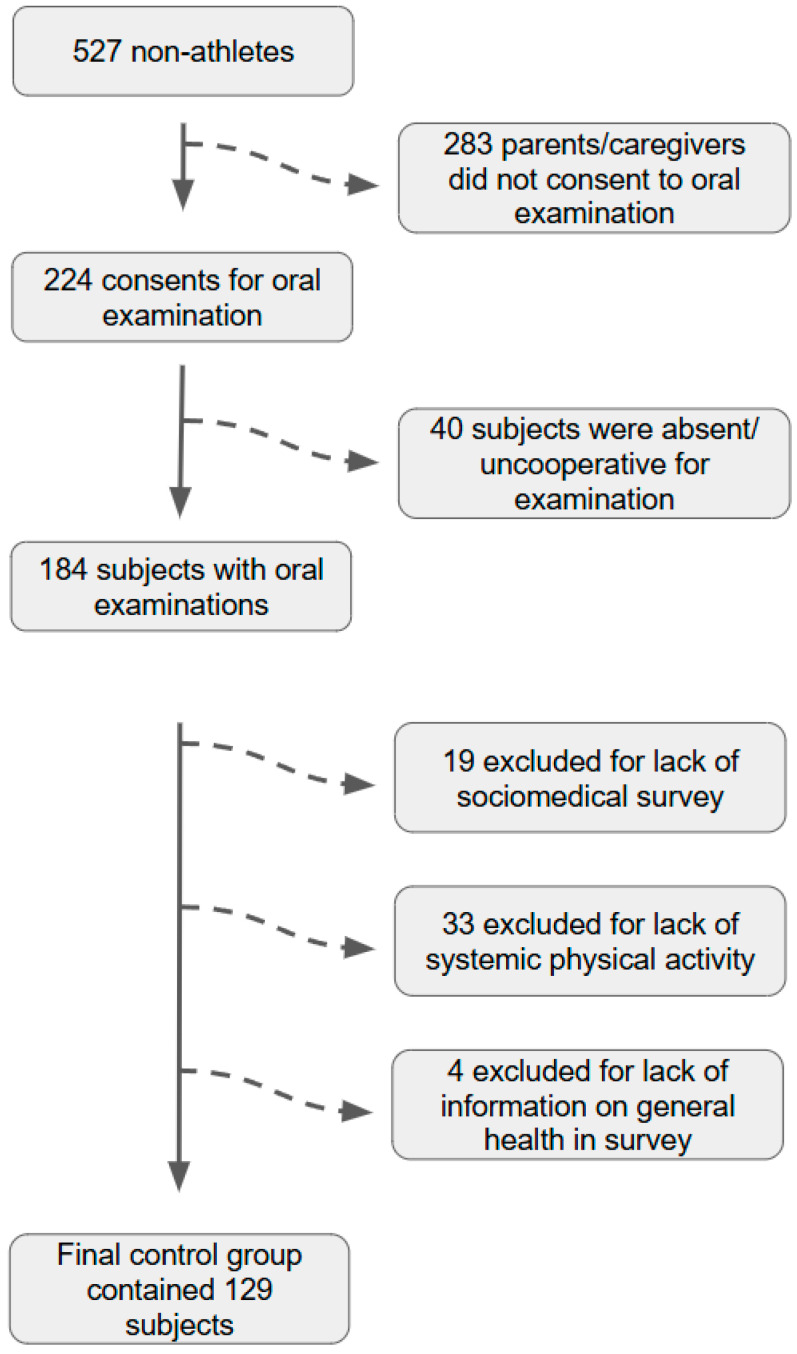
A flow diagram explaining how the final control group was selected based on the initial group of non-athletes who met all of the inclusion criteria.

Regarding the control group, it was estimated that 527 students who do not actively practice sports and are not Special Olympics members attended these schools (Figure 2). The same rules as in the study group were applied. There were 224 consent forms and 205 completed sociomedical surveys collected. However, only 184 children had dental examinations performed because 40 students were not at school during each visit or were uncooperative. In the end, 56 children were also excluded from further analysis due to the lack of a completed sociomedical survey (19 students), the lack of some systematic physical activity (33 students), or the lack of information concerning general health in the sociomedical survey (4 students).

Finally, we included 100 subjects in the study group and 128 of their counterparts in the control group. The inclusion criteria for the subjects in the study and control groups in the research are as follows:

Study group:

- Practicing a sport as a Special Olympics member at a special needs school under the supervision of a trainer;
- Information in the sociomedical survey related to the discipline of sport that is practiced by the child.

Control group:

- Not practicing a sport at a special needs school under the supervision of a trainer, or other intensive physical activity.

Study and control group:

- Parental written and informed consent;
- Child’s cooperation;
- Completed sociomedical surveys by parents/caregivers;
- All answers to the questions concerning traumatic injuries in the sociomedical survey returned by parents;
- Information in the sociomedical survey related to the sport practiced by the child:
- Subjects from six special needs schools situated in Poznan and one school in Szamotuly;
- Subjects residing in Wielkopolska province;
- Subjects present during the training or at school during the days on which the examinations were performed:
- Groups with the same ethnic, cultural, demographic, or regional origin;
- Age between 8 and 30 years old;
- A properly completed sociomedical survey by the parent/caregiver and the patient’s dental chart.

### 2.2. Sociomedical Survey of Parents/Caregivers

As a part of the research, a sociomedical study was performed among the parents/legal caregivers of athletes and students from the control group. The surveys were distributed by trainers and teachers during parent–teacher meetings, and respondents filled them in at home. Participation in the study was voluntary, and full confidentiality of the collected data was ensured for the participants.

A self-designed sociomedical survey was developed and made available in paper format. Close-ended questions concerned the following:

- Information on the person that filled in the survey;
- Formal education of the mother and father;
- The type of residence of the child;
- The type of disability of the individual;
- The level of his/her disability;
- Other coexisting disorders;
- The discipline of sport that the child is practising (What kind of sport does your child practice?);
- The number of years of playing sports (How many years has your child been playing sports?);
- The number of hours of training per week (How many hours a week does your child play sports?);
- The use of a mouthguard (Does your child use a mouthguard?).

Parents/caregivers whose children had ever suffered from a traumatic injury were also asked to answer a few additional questions referring to the following:

- The place/situation where the child was injured (What were the circumstances of the injury?);
- The type of injury (What type of injury was it?).

The athletes played different sport activities which were grouped for this study into the following categories: athletics (track and field), including shot put, long jump, and running; cycling with bicycles and three-wheeled bikes (tricycles); and winter sports including skiing and cross-country skiing, according to the Special Olympics rules [19]. Moreover, the participants also practiced Bocce, speed skating/skating, table tennis, swimming, football, bowling, judo, and MATP. Most of the athletes practiced more than one sports discipline.

The indicators of reliability were determined using internal consistency reliability and the scale’s test–retest reliability. The test–retest reliability survey was carried out among 10 additional parents who completed the sociomedical survey twice at 2-week intervals. The values of the coefficients of reliability for the test–retest amounted to 0.83. The coefficient of reliability for internal consistency using Cronbach’s Alpha was 0.89.

### 2.3. Information from the Sociomedical Surveys Concerning the Degree of Intellectual Disability and the General Diseases of the Subjects

The degree of intellectual disability of special needs students is normally assessed by specialists in psychology of public psychological and educational counselling centres who are members of assessment boards [20]. Based on documents delivered by parents/caregivers from other specialists, medical professionals (including physicians) give their opinion concerning the child’s physical or sensory disability. Under the advice of those specialists, special needs students are allocated to a particular category of students, i.e., classes.

### 2.4. Dental Evaluation

The parents/legal caregivers signed the consent forms for their children to participate in the examination. Consent was obtained from a parent or guardian of both juvenile and adult athletes.

The students took part in the dental evaluation voluntarily. The examination was performed using the “tell, show, do technique” and positive reinforcement, without any physical restraints or pharmacological sedation. The procedure was not carried out if the individual failed to cooperate or refused to participate.

The orofacial examination was conducted with the use of an overhead lamp, a ball-ended dental explorer, and a plain mouth mirror, with disposable masks and gloves.

The clinical examination was carried out in the nurse’s office of a special needs school. The subject was seated in a chair with his/her head resting against the wall; however, in some cases, the nurse or teacher were needed to help stabilize their head. The dentition was inspected while wet without any previous professional cleaning. In some cases, a cotton roll was utilized to remove the debris from the tooth surface.

The oral examination was conducted by two experienced, independent examiners to minimize potential bias and inaccuracies associated with a single examiner.

Prior to the examination, the examiners (A.H.-P. and P.P.) were trained and assessed by another experienced dentist, a specialist in paediatric dentistry (K.G.), according to the World Health Organization (WHO) manual for training providers [21]. Training also included instruction concerning the completion of the examination form that was self-designed.

The intra-examiner and inter-examiner agreement for traumatic injuries was assessed by another recall visit of 10 patients after two weeks, with a κ that amounted to 0.96 and 0.92, respectively.

Using specially designed data collection forms, recorders marked the results of the oral examination by noting the following: no sign of injury (0); extensive restoration suggesting treated injury (1); crown fracture within enamel (2); crown fracture within enamel and dentine (3); crown fracture with pulp involvement (4); tooth missing due to trauma (5); discoloured tooth indicating loss of pulpal vitality (6); other damage. Additionally, the extent/degree/status of traumatic injury was marked on pictures of the teeth in the patient’s chart. The severity of dental trauma was measured in terms of the number of teeth affected.

Moreover, the dentists also carried out extra- and intraoral evaluations to diagnose traumatic injury of soft tissues. Examiners marked the results by noting the following: no sign of injury (0); injury of the lips (1); injury of the tongue (2); injury of the gingiva (3); injury of the cheek (4); injury of the chin (5); injury of the eyebrows (6); injury of the forehead (7); injury of the nose (8); other injury (9).

Restorations on anterior teeth—primarily incisal and either diagonal or horizontal in shape—were assessed as those placed because of dental injury, as in the Special Olympics Special Smiles examination [18]. Teeth missing due to caries (MT) were not taken into account. The data concerning missing and restored teeth were verified by interview. Otherwise, the tooth was not included in the analysis.

The number of single permanent teeth with developmental enamel defects, such as enamel opacity or hypoplasia, in the anterior region of the dental arch in the subjects was evaluated. The tooth was not taken into consideration for the analysis if any other tooth in the oral cavity had such changes, since they could be caused by factors other than traumatic injury to the deciduous predecessor.

After the oral examination, each athlete received oral hygiene instructions. The patients’ parents/legal caregivers were provided with written information concerning the oral health status and dental treatment needs of their child. An address of a dental school, where they might report to for dental treatment if they had no family dentist, was also provided. Moreover, the dentists advised the use of mouthguards among athletes who participated in all sports disciplines that would place them at risk of orofacial injuries.

Based on the collected data, the researchers evaluated the number of teeth with traumatic injuries, the number of teeth with opacities and hypoplasia, the number of teeth with traumatic injuries in particular subjects, the number of particular teeth affected by trauma, and the number of patients with dental and soft tissue injuries in the study and control groups. The number of teeth treated due to traumatic injuries, as well as those that need such therapy, was also assessed.

### 2.5. Statistical Analysis

Data from the study were coded and entered by the researchers into an Excel spreadsheet, and then double-checked to verify their accuracy. Statistical analysis, with the use of the difference test between two proportions and Statistica Software v.10 (StatSoft Inc., Tulsa, OK, USA), took into consideration the number of subjects with traumatic injuries to the teeth and soft tissues of the face and oral cavity, as well as the number of anterior teeth (incisors and canines) with traumatic injury concerning the dental arch (maxillary and mandibular), in the study and control groups, as well as the differences between genders. A comparison between the variables, including traumatic injuries to the teeth, the number of teeth affected in schoolchildren, and the number of affected teeth in relation to age, sex, degree of intellectual disability, presence of physical and/or sensory disability, discipline of sport, general disorder, and mothers’ and fathers’ level of schooling/education, was conducted using a Chi-square test. A value of *p* ≤ 0.05 was considered statistically significant.

## 3. Results

The characteristics of respondents of the sociomedical survey who care for the special needs students in the study and control groups are presented in Table 1.

A total of 358 individuals were initially recruited, 130 of whom were disqualified, while 228 completed the study. The inability to obtain a completed sociomedical survey by parents/caregivers and a dental examination of the participant was the reason for disqualification. The demographic characteristics of the participants are presented in Table 2.

The final study group included 100 subjects (33% females and 67% males), aged from 8 to 30 years old (the mean age and standard deviation were 17.26 ± 4.67) (Table 2). A total of 3 subjects (3%) were under 10 years of age, 27% between 10 and 14 years, 42% between 15 and 20 years, 25% between 21 and 25 years, and 3% over 25 years old. Ninety-seven subjects were students of a special needs school, and three individuals attended occupational therapy workshops at those schools. The control group was composed of 128 counterparts (59 females—46% and 69 males—54%) that did not play any sport, aged from 8 to 25 years (14.41 ± 4.46). A total of 16 subjects (12%) were under 10 years of age, 48% between 10 and 14 years, 27% between 15 and 20 years, and 13% between 21 and 25 years.

Out of 100 parents/caregivers of subjects practicing sport, 21% respondents did not give any information concerning the general disease or other accompanied disorders of their child and 27% individuals wrote that their child did not have any other disorder except an intellectual disability; meanwhile, 17% of parents/caregivers informed us that their child had Down syndrome, 22% had cerebral palsy and/or epilepsy, 4% had attention deficit hyperactivity disorder (ADHD) and/or aggressive behaviour, 3.0% had autism, 2% schizophrenia, and a single subject each had Angelman syndrome, Arnold Chiari syndrome, fragile X syndrome, or congenital malformation syndrome. In total, 23% of subjects had a mild, 48% moderate, 28% severe, and 1% profound intellectual disability (Table 2).

Control group subjects had 1010 permanent and 3 primary incisors as well as 394 permanent and 105 primary canines, with 42 subjects who had mixed dentition and 86 who had permanent dentition. Individual subjects had from 5 to 12 permanent and from 0 to 4 primary anterior teeth present in the oral cavity.

In the total group of study subjects, sixteen individuals had 18 teeth with signs of traumatic injuries, whereas in four persons, six permanent teeth with opacities or hypoplasia were observed that might be sequelae of traumatic injury to the primary predecessor. Out of 18 injured teeth, 17 (94%) involved the maxillary permanent incisors. Statistically significant differences were observed between maxillary and mandibular permanent incisors and all teeth of the anterior region of the dental arch in relation to teeth with traumatic injuries and those with enamel opacities (*p* < 0.001 and *p* = 0.046, *p* = 0.006 and *p* < 0.0001, respectively) (Table 3). When comparing the study and control groups, differences were seen between primary canines and total primary teeth of the anterior region of the dental arch with traumatic injuries (in both, *p* = 0.0005).

The prevalence of traumatic injuries among soft and hard tissues in the study group was 18%, whereas among teeth it was 16%; in the control group, the values were 13% and 10%, respectively.

The most common scenario comprised having one tooth affected by traumatic injury (in study, 14%; in control, 5%), and the difference between both groups was statistically significant (*p* = 0.02) (Table 4).

Mainly maxillary left (tooth 21) and right (tooth 11) central incisors were affected by trauma, in both the study and control group, with results amounting to 8% and 7% for the study group and 5% and 7% for the control group, respectively. A statistically significant difference was observed between females (9%) and males (22%) of the study group in relation to the number of teeth with traumatic injuries (*p* = 0.01). Individual persons had other anterior teeth affected by injury, i.e., the upper right permanent lateral incisor (tooth 12), upper left permanent lateral incisor (tooth 22), lower left permanent central incisor (tooth 31), lower right permanent central incisor (tooth 41), and upper right primary canine (tooth 53). In two females (3%) in the control group, the lower right permanent lateral incisors (tooth 42) were damaged by traumatic injury.

In both groups, fractures within the dental crown were the most common types of injuries (11% and 10%, respectively), and included fractures within enamel (2% in both groups) as well as within the enamel and dentine (9% and 8%, respectively). Injuries to soft tissues included areas such as the lips (two persons in the study group and one individual in the control group), tongue and gingiva (individual participants in the study group), chin and eyebrow (individual persons in the control group), and forehead (two people in the study group).

Dental evaluation revealed that 6 (37%) males out of the total 16 subjects affected by dental injuries from the study group still needed some restorative procedures after trauma at the time of examination. These males had untreated fractured teeth, which should be restored. Six athletes (37%), including three females (100%) and three males (33%), had dental treatment previously performed. Regarding the treatment procedures that were previously completed, most were crowns with fractures that were restored (19%), endodontic treatment in one case (6%), one subject (6%) had splinted teeth, and one (6%) had dental extraction performed.

In 11 controls (85%), necessary dental treatment due to traumatic injury was observed. All of them needed crowns restored. Only two students (15%, two males) had dental treatment performed before our examination.

The parent of fourteen students out of eighteen students actively practicing sport gave information concerning the place where the orofacial traumatic injury happened. Only 3 (17%, one female and two males) athletes were injured during training or competition, 10 (56%, three females and seven males) at school or home, and 1 male (6%) was injured in some other type of situation. Out of the whole group of athletes that had traumatic injuries to soft and hard tissues, 9 (50%) subjects trained up to 2 h, 4 (22%) between 2 and 3 h, and 5 (28%) students trained over three hours per week.

Additionally, analysis of the association between variables related to traumatic injuries to the teeth and those concerning the children’s and parents’ characteristics was carried out; however, it did not reveal any correlation.

In the total study group, out of 18 subjects, 13 (72%) had only dental traumatic injury, 3 (17%) had injury to dental and soft tissues, and 2 (11%) individuals had only injury to soft tissues.

## 4. Discussion

To the best of our knowledge, this is the first complex report concerning the orofacial injuries of athletes with intellectual disabilities and other coexisting general diseases residing in the Wielkopolska province in Poland who exercise intensively and participate in different sports tournaments. All athletes are from the same environment, attending the same special needs schools, and they are coached by physical education teachers at those educational institutions.

The results of the present study show that the most prevalent type of injury is a crown fracture of the maxillary central incisors, since in both groups, crown fracture within the enamel and dentin was the most frequent, at 9% and 8%, respectively; fracture within the enamel that amounted to 2% in both the study group and the control group. This is in agreement with other scientific research concerning the aetiology of traumatic dental injuries in permanent dentition [8,16]. Moreover, maxillary central incisors were the most commonly affected, as in previous studies concerning individuals with intellectual disabilities [12].

The results of other studies carried out among special needs patients, i.e., with an intellectual disability and/or other general disorders, including athletes from such a population, may be somewhat different. However, Dagon et al. performed a study in a population of two hundred and forty-nine athletes, aged between 10 and 65 years (mean 29.20 ± 11.24 years), with special needs who attended the 2016 Israeli Special Olympics games [8]. The authors revealed the occurrence of dental trauma among as many as 27.30% of participants. Moreover, 17.67% of the cases were categorized as severe, whereas 9.64% were categorized as mild. Most of the traumas involved the maxillary incisors (94%), and this corroborates the results of our study (94%). Nevertheless, it must be emphasized that the individuals who participated in the Israeli dental examination arrived voluntarily according to different games’ schedules, were not recruited by dentists, and played different sports activities. It must be emphasized that in our study, the researchers examined all cooperating children whose parents/caregivers gave their informed consent in both the study and control group. On the other hand, Dagon et al. found that the ratio of dental trauma was similar for females and males (28% and 26%, respectively), with no statistically significant differences. However, in their research, there were no data concerning the specific disabilities of the athletes, in contrast to our study. Moreover, we observed that dental traumatic injuries were seen more often in males (22%) than in females (9%), which is also described in the literature [21,22]. Dellavia et al. performed a study in athletes of the Italian Special Olympics National Games and revealed a lower prevalence (5.9%) of traumatic injuries to anterior teeth than in our research [23].

Moreover, some studies that were performed among a population of intellectually disabled athletes in numerous countries present results concerning dental traumatic injuries. For example, Fernandez et al. revealed that the prevalence of injury to the teeth was 13.02% of 15,941 individuals (aged 28.5 ± 5.9 years) from 49 countries of Europe and Eurasia [10]. Interestingly, there were vast discrepancies between the results, and the highest prevalence was in Poland (25.73%), Cyprus (25.00%), and Switzerland (23.91%), whereas the lowest was in Montenegro (0.00%), Kyrgyz Republic (0.00%), and Armenia (4.11%). However, as the researchers suspected, some of the athletes may have been be examined a few times during different competitions since the examination forms were anonymous and could not be verified. In addition, the authors found out that the leading causes of traumatic dental injuries were falls during athletic competitions, whereas in the population of the study group from Poznan, the principal reason for traumas was accidents at home or school, while only three athletes suffered from trauma during training or competitions.

The null hypothesis in this study is rejected since in special needs students from Wielkopolska, there were no differences in the prevalence of traumatic injuries to the teeth and soft tissues between the study group and controls. In athletes, such injuries might be sports-related. However, they could be caused, as in other individuals with intellectual disabilities and other coexisting general diseases, by additional risk factors such as seizures, coordination difficulties, incompetent lips, slow reflexes, or increased overjet of maxillary incisors [10]. Interestingly, in Polish epileptic patients, oral traumatic injuries occurring during seizures were found in 62% of individuals and mainly affected the lips, cheek, and tongue [14]. In the population of special needs students with an intellectual disability and epilepsy, 15.9% of subjects had crown fractures, whereas in the control group without epilepsy, crown fractures were noted in 6.4% of individuals [12].

Traumatic injuries to the primary teeth may be a causative agent compromising the development of permanent successors [20]. The sequelae to permanent dentition caused by traumatic injuries to their deciduous predecessors are described as enamel hypoplasia, white or yellow-brown discoloration of enamel, crown or root dilaceration, root duplication, odontoma-like malformation, partial or complete arrest of root formation, eruption disturbances, and sequestration of the permanent tooth germ [24]. However, enamel hypoplasia and discoloration of enamel are the most common sequelae [24]. Therefore, we decided to include in the examination and analysis singular permanent teeth with such changes. Our study revealed enamel opacities in 5 (0.64%) incisors of the individuals in the study group and in 11 (1.09%) of the control group, whereas hypoplasia was seen in 0.13% and 0.10%, respectively. Other researchers also observed developmental defects in single permanent anterior teeth in a population of patients with intellectual disabilities and chronic diseases. Dubey et al. performed a study among seventy children and adolescents, aged between 7 and 18 years old, with cerebral palsy in India [25]. Their results revealed enamel hypoplasia of a single tooth in two cases (2.8%). These children reported primary tooth avulsion due to a fall in the past. However, we do not have information from the interview or sociomedical survey of parents/caregivers confirming traumatic injuries to deciduous dentition. Therefore, it is only our supposition. In addition, Modric et al., in their study carried out in a group of seventy-two children with intellectual disabilities, aged from 5 to 18 years (mean 12.44 ± 3.65 years), found eight children with single permanent tooth affected by developmental defects of enamel and seven children with two permanent teeth with such changes [26]. Alhammad examined one hundred and forty children with cerebral palsy in Riyadh, Saudi Arabia [27]. The study group consisted of individuals between 3 and 12 years (82 males and 58 females). They found four children with a labial defect of enamel of the permanent central incisor. In three cases, the upper incisor was affected, and in one case, the lower incisor was affected.

In most papers concerning dental traumatic injuries in individuals with intellectual disabilities, there is a shortage of combined relevant data, such as the type of injury, including soft and hard orofacial tissues, teeth with a higher prevalence of trauma, whether the tooth was fractured, avulsed, or discoloured, and suspected sequelae of traumatic injuries of primary teeth to the permanent successors. Therefore, it would be necessary to develop and use a detailed screening protocol. Moreover, it would be beneficial if the studies also gave information on sociodemographic data concerning patients and their caregivers.

It must be emphasized that during the Special Olympics oral health-related screening, the standardized Special Olympics Special Smiles (SOSS) protocol is normally used to evaluate the oral health of athletes. However, Fernandez et al. noticed that oral health data obtained from the studies of special athletes must be interpreted cautiously since the data collection form used at SOSS screening events is not the same as those used for general population research [28]. The authors observed that a need exists for developing a data collection form that could be compared more closely to those that are utilized to collect data among the general population. Therefore, in the present study, we used a much more detailed form and questionnaire for parents/caregivers.

Interestingly, in the current study, we did not observe any association between variables related to traumatic injuries to the teeth and those concerning the children’s and parents’ characteristics. Freire et al. noted that boys attending schools situated in lower socioeconomic districts and those whose mothers had the lowest level of education had a higher chance of having two or more teeth with traumatic dental injuries [16]. Substantially, it must be remembered that athletes with intellectual and physical disabilities are a higher functioning and supported stratum of the individuals in comparison to their non-athlete counterparts with disabilities [28,29]. Therefore, they might be much more agile, and for that reason, avoid traumatic injuries.

We might assume that most of the athletes reported to the dental surgery with their parents for an emergency visit after a traumatic injury (69%); however, not all of the teeth were treated due to the lack of cooperation of patients.

Normally, the outcome of dental trauma treatment is highly related to the skills and knowledge of the dentist and to the first aid provided at the place of the accident [9]. Therefore, not only medical professionals, including dentists, but also parents/caregivers, coaches, and teachers should have basic knowledge concerning emergency management of such cases. A person with traumatic injury to a tooth becomes a challenge for dentists because of the uncertainty of the treatment prognosis and the peculiarity of the situation [9]. The situation is much more serious when the traumatic injury affects the teeth of individuals with intellectual disabilities due to, among other things, problems of cooperation with the patient. In addition, such a population also faces numerous barriers in access to systematic and planned dental care, including social, financial, and physical hindrances [30,31,32,33,34]. It needs to be emphasized that a dentist’s decision in providing care for patients with special needs is dictated by a multiplicity of factors, including behavioural, time, financial, and educational constraints [32].

Moreover, treatment of teeth after traumatic injury, in healthy as well as disabled patients, may be a challenge for the majority of practicing dentists since it is not a routine and ordinary procedure. The treatment demands proper cooperation with the patient, adequate emergency management, accurate diagnosis, and correct treatment with follow-up visits [9].

It needs to be emphasized that treatment of traumatic dental injury in a juvenile patient is often complicated, unpredictable, expensive, and could have an impact on the rest of the patient’s life. Therefore, the consequences of dental trauma might have a lifelong impact on an individual’s quality of life [9].

Therefore, special needs patients are a group in particular need of preventive dental care [34]. It must be emphasized that the procedures should include prevention of oral diseases as well as dental and oral traumatic injuries. Therefore, dental professionals should encourage the use of mouthguards among athletes participating in organized sports, especially those with intellectual disabilities who practice contact sports [29]. The number and severity of orofacial injuries might be significantly reduced by the use of mouthguards [29]. Such devices protect periodontal and dental tissues during contact sports and lessen the number and severity of injuries. However, the present study revealed that only one special needs athlete utilizes such a device during training and competitions. We may speculate that such a situation might be caused by a lack of knowledge among the athletes’ caregivers concerning the use of mouthguards in general. Therefore, it must be emphasized that there is an urgent need for education regarding preventive measures against traumatic injuries among parents/caregivers and trainers of special needs students who actively practice sports.

Intriguingly, Suzuki et al. provided custom mouthguards to 503 athletes, with a mean age of 23.8 years, who desired them, out of 930 participants of the Special Smiles project of the Healthy Athletes program during the Eighth Special Olympics World Winter Games in Nagano [35]. The mouthguards were delivered to the athletes, in addition to floor hockey players who were more likely to use them. Interestingly, the preparation of the mouthguard lasted approximately one hour. It was made of 2 mm single-layered ethylene-vinyl acetate (EVA) and shaped with a vacuum former [35]. Notwithstanding, the authors noticed that some athletes seemed to have slight breathing difficulties as they were not used to mouthguards. Therefore, they suggested to actively promote the use of mouthguards among individuals practicing sports so they can get comfortable with their use before competitions.

Patients who have suffered a traumatic dental injury report decreased oral health-related quality of life [15]. Needless to say, they might also suffer from lower self-esteem because of their appearance, mainly where the injury is not efficiently managed [15].

We are aware that the present study has some potential limitations. Firstly, radiographs of the traumatized teeth were not taken since the survey was carried out at a school that was not equipped with imaging equipment. Moreover, students’ parents is not have provided the full medical history concerning the general disease of their child, as well as data on the individual’s characteristics. In addition, another limitation was that not all of the athletes and other special needs students took part in the dental examination due to a lack of parental consent and a lack of cooperation. Furthermore, it is very difficult to compare the results of this study with those of other studies since they differ in methodology, trauma classification, and the dentition studied [10].

However, several strengths of this study must be acknowledged. Firstly, the sample size was large; therefore, the special needs athletes may represent the wider population of individuals with an intellectual disability who practice sports in Wielkopolska province and Poland, and the conclusions drawn from this population may be objectively interpreted. In addition, there were two examiners involved in the study who were trained and assessed prior to the research to prevent the possible bias and inaccuracies that could be introduced by a single examiner. The examination was carried out in a homogeneous group of special needs athletes, and their counterparts attending the special needs schools were from the same region. Moreover, the analysis included valuable information from the sociomedical survey study completed by parents. Additionally, in this study, numerous data were included, such as the prevalence and severity of traumatic damage to the teeth, the number of teeth with trauma, the type of injury, and also a wide range of characteristic of two groups of patients with an intellectual disability, i.e., one practicing sports and the other undertaking no physical activity.

The authors of the studies carried out among populations with higher numbers of athletes who participated in Special Olympics competitions, and very often in different countries, noticed that some individuals may participate in a few events during the time of collecting the data [10]. Therefore, bias in data gathering might be involved. Thus, it needs to be emphasized that in the present study, all subjects from the study and control groups were examined only once. Moreover, the study and control groups were from the same environment, i.e., individuals attended the same schools and they were living in the same region (Wielkopolska province), which is another strength of our research.

Interestingly, analysis of the association between variables related to traumatic dental injuries and those concerning the children’s and parents’ characteristics did not reveal any correlation. Moreover, according to the data from the sociomedical survey completed by parents, most traumatic injuries in students actively practicing sports happened at home or school, as in the control group, since only three athletes experienced trauma during training or competitions.

### Summary and Perspective

Because of the relatively high percentage of dental trauma in athletes with intellectual disabilities in the present study and in Special Olympics participants in other studies [8], there is an urgent need to promote the use of mouthguards in this population during both training and competition, necessitating previous familiarization with such equipment among the individuals. Moreover, it is necessary to create special materials on this topic and introduce education programs for the trainers/coaches, teachers, and therapists working in special needs schools and in other institutions teaching athletes with intellectual disabilities. Additionally, such materials and courses should also be prepared for parents/caregivers/guardians, dentists, and physicians who look after this population to increase their awareness concerning dental traumatic injuries and the ways to prevent them.

Needless to say, there is a need to educate parents/caregivers as well as trainers and coaches about the systematic use of mouthguards at a young age, as athletes should become accustomed to them [36].

Additionally, the results of the present study highlight the need to design a standardized data collection form that could be used to collect data and compare oral health status between individuals with disabilities and chronic disorders and the general population.

## 5. Conclusions

The present study highlights an urgent need for preventive programs targeting athletes with intellectual disabilities and other coexisting conditions, as well as educational courses for their parents, caregivers, and trainers on the use of mouthguards during contact sports. Dentists should be professionally prepared to meet the special needs of this particular population to minimize the burden of dental trauma. The findings also highlight the need to design a standardized and unified data collection form that could be used for comparison of oral health status, including traumatic injuries, between people with disabilities and the general population.

## Figures and Tables

**Table 1:** Characteristics of respondents.

Respondents’ Characteristics		Study Group			Control Group			Total		
		Females *n* = 33	Males *n* = 67	*p* Females vs. Males	Females *n* = 59	Males *n* = 69	*p*	Study Group *n* = 100	Control Group *n*= 128	*p*
The sociomedical survey was completed by:	mother *n* (%)	22 (67%)	49 (734%)	0.47	46 (79%)	62 (90%)	0.06	71 (71%)	108 (85%)	0.02 *
	father *n* (%)	5 (15%)	6 (9%)	0.28	5 (8%)	4 (6%)	0.65	11 (11%)	9 (7%)	0.29
	mother and father *n* (%)	0 (0%)	4 (6%)	0.15	2 (3%)	2 (3%)	0.72	4 (4%)	4 (3%)	0.68
	aother person *n* (%)	6 (18%)	7 (10%)	0.26	6 (10%)	1 (1%)	0.02 *	13 (13%)	7 (5%)	0.03 *
	no answer *n* (%)	0 (0%)	1 (2%)	0.56	0 (0%)	0 (0%)	1.00	1 (1%)	0 (0%)	0.26
Formal education of the mother	primary *n* (%)	10 (30%)	16 (24%)	0.52	6 (10%)	6 (9%)	0.69	26 (26%)	12 (9%)	<0.001 *
	vocational *n* (%)	4 (12%)	7 (10%)	0.76	9 (15%)	9 (13%)	0.74	11 (11%)	18 (14%)	0.49
	secondary *n* (%)	12 (37%)	23 (34%)	0.84	24 (41%)	34 (49%)	0.36	35 (35%)	58 (45%)	0.13
	university *n* (%)	7 (21%)	18 (27%)	0.51	12 (20%)	18 (26%)	0.42	25 (25%)	30 (24%)	0.75
	no answer *n* (%)	0 (0%)	3 (5%)	0.24	8 (14%)	2 (3%)	0.03 *	3 (3%)	10 (8%)	0.18
Formal education of the father	primary *n* (%)	8 (24%)	11 (16%)	0.33	5 (8%)	8 (12%)	0.56	19 (19%)	13 (10%)	0.052
	vocational *n* (%)	3 (9%)	8 (12%)	0.64	18 (31%)	17 (25%)	0.53	11 (11%)	35 (27%)	0.003 *
	secondary *n* (%)	13 (40%)	28 (42%)	0.77	18 (31%)	31 (45%)	0.10	41 (41%)	49 (38%)	0.64
	university *n* (%)	5 (15%)	15 (23%)	0.41	6 (10%)	10 (14%)	0.49	20 (20%)	16 (13%)	0.09
	no answer *n* (%)	4 (12%)	5 (7%)	0.40	12 (20%)	3 (4%)	0.004 *	9 (9%)	15 (12%)	0.47

*n*—number of subjects; * *p* ≤ 0.05—statistically significant value.

**Table 2:** Characteristics of individuals from study and control groups.

Patients Characteristics		Study Group			Control Group			Total		
		Females *n* = 33	Males *n* = 67	*p* Females vs. Males	Females *n* = 59	Males *n* = 69	*p* Females vs. Males	Study Group *n* = 100	Control Group *n* = 128	*p* Study vs. Control Group
Age of subjects in years	(mean ± SD)	17.7 ± 4.9	17.0 ± 4.6		13.7 ± 4.2	15.0 ± 4.6		17.3 ± 4.7	14.4 ± 4.5	
	min–max	8–28	9–30	0.48	8–23	9–25	0.09	8–30	8–25	<0.001 *
	median	18	17		13	14		17	13	
Physical disability	yes *n* (%)	17 (51%)	28 (42%)	0.39	29 (49%)	38 (55%)	0.49	45 (45%)	67 (52%)	0.29
	no *n* (%)	16 (49%)	39 (58%)	0.34	30 (51%)	31 (45%)	0.49	55 (55%)	61 (48%)	0.29
Type of disability	intellectual	15 (46%)	37 (55%)	0.35	24 (41%)	22 (32%)	0.35	52 (52%)	46 (36%)	0.01 *
	intellectual and physical	17 (51%)	26 (39%)	0.22	23 (39%)	33 (48%)	0.25	43 (43%)	56 (44%)	0.91
	intellectual and sensory	1 (3%)	2 (3%)	0.91	6 (10%)	9 (13%)	0.59	3 (3%)	15 (12%)	0.01 *
	intellectual, physical, and sensory	0 (0.00)	2 (3%)	0.31	6 (10%)	5 (7%)	0.54	2 (2%)	11 (8%)	0.046 *
Degree of intellectual disability	mild *n* (%)	5 (15%)	18 (27%)	0.18	22 (37%)	27 (39%)	0.82	23 (23%)	49 (38%)	0.01 *
	moderate *n* (%)	20 (61%)	28 (42%)	0.09	25 (43%)	23 (33%)	0.29	48 (48%)	48 (37%)	0.09
	severe *n* (%)	8 (24%)	20 (30%)	0.59	10 (17%)	16 (23%)	0.32	28 (28%)	26 (21%)	0.16
	profound *n* (%)	0 (0%)	1 (1%)	0.56	2 (3%)	3 (5%)	0.76	1 (1%)	5 (4%)	0.30
Type of residence	living at home with parents *n* (%)	26 (79%)	61 (90%)	0.07	51 (87%)	66 (96%)	0.08	87 (87%)	117 (91%)	0.33
	with grandparents without parents *n* (%)	0 (0%)	1 (2%)	0.56	2 (3%)	2 (3%)	0.71	1 (1%)	4 (3%)	0.29
	caring institution (orphanage) *n* (%)	5 (15%)	3 (4%)	0.051	6 (10%)	0 (0%)	0.007 *	8 (8%)	6 (5%)	0.19
	boarding school, during holidays at home *n* (%)	2 (6%)	1 (2%)	0.14	0 (0%)	1 (1%)	0.55	3 (3%)	1 (1%)	0.27
	no answer *n* (%)	0 (0%)	1 (2%)	0.56	0 (0%)	0 (0%)	10.00	1 (1%)	0 (0%)	0.26
Other coexisting disorders	cerebral palsy and/or epilepsy *n* (%)	8 (24%)	14 (21%)	0.65	23 (39%)	22 (32%)	0.48	22 (22%)	45 (35%)	0.03 *
	Down syndrome *n* (%)	4 (12%)	13 (19%)	0.37	1 (2%)	5 (7%)	0.09	17 (17%)	6 (5%)	0.001 *
	attention deficit hyperactivity disorder (ADHD) and/or aggressive behaviour *n* (%)	0 (0%)	4 (6%)	0.15	1 (2%)	4 (6%)	0.19	4 (4%)	5 (4%)	0.68
	autism *n* (%)	0 (0%)	3 (5%)	0.24	6 (10%)	13 (19%)	0.19	3 (3%)	19 (15%)	0.002 *
	schizophrenia *n* (%)	2 (6%)	0 (0%)	0.04	2 (3%)	1 (1%)	0.41	2 (2%)	3 (2%)	0.86
	other rare syndromes *	1 (3%)	3 (5%)	0.80	4 (7%)	1 (1%)	0.11	4 (4%)	5 (4%)	0.96
	intellectual disability	12 (37%)	15 (22%)	0.14	12 (20%)	14 (21%)	0.98	27 (27%)	26 (20%)	0.21
	no answer	6 (18%)	15 (22%)	0.64	10 (17%)	9 (13%)	0.63	21 (21%)	19 (15%)	0.24
**Characteristics of Patients from Study Group (Cont.)**					**Females** ***n* = 33**	**Males** ***n* = 67**	***p*** **Females vs. Males**		**Total** ***n* = 100**	
Discipline trained by the player		athletics (track and field) *n* (%)			13 (40%)	21 (31%)	0.42		34 (34%)	
		cycling *n* (%)			1 (3%)	9 (13%)	0.11		10 (10%)	
		bocce *n* (%)			7 (21%)	5 (7%)	0.04 *		12 (12%)	
		winter sports (skiing, cross country skiing) *n* (%)			4 (12%)	11 (16%)	0.60		15 (15%)	
		Ice-skating/skating *n* (%)			5 (15%)	2 (3%)	0.03 *		7 (7%)	
		table tennis *n* (%)			1 (3%)	14 (21%)	0.02 *		15 (15%)	
		swimming *n* (%)			10 (30%)	23 (34%)	0.68		33 (33%)	
		football *n* (%)			4 (12%)	16 (23%)	0.16		20 (20%)	
		bowling *n* (%)			4 (12%)	2 (3%)	0.07		6 (6%)	
		judo *n* (%)			4 (12%)	10 (15%)	0.68		14 (14%)	
		MATP (Motor Activity Training Program) *n* (%)			4 (12%)	6 (9%)	0.63		10 (10%)	
Years of playing the sport		up to 2 years *n* (%)			7 (21%)	15 (22%)	0.90		22 (22%)	
		over 2 up to 6 years *n* (%)			15 (46%)	26 (39%)	0.50		41 (41%)	
		over 6 up to 10 years *n* (%)			3 (9%)	13 (19%)	0.19		16 (16%)	
		over 10 up to 14 years *n* (%)			2 (6%)	4 (6%)	0.83		6 (6%)	
		over 14 years *n* (%)			3 (9%)	3 (5%)	0.31		6 (6%)	
		no answer *n* (%)			3 (9%)	6 (9%)	0.86		9 (9%)	
Hours of training the sport per week		up to 2 h			22 (67%)	36 (54%)	0.22		58 (58%)	
		over 2 up to 3 h			10 (30%)	13 (19%)	0.21		23 (23%)	
		over 3 h			1 (3%)	18 (27%)	0.005 *		19 (19%))	
Wearing a mouthguard while playing the sport		yes, always during training and competitions *n* (%)			0 (0%)	1 (2%)	0.56		1 (1%)	
		yes, only during competitions *n* (%)			0 (0%)	0 (0%)	1.00		0 (0%)	
		yes, only during training *n* (%)			0 (0%)	0 (0%)	1.00		0 (0%)	
		sometimes *n* (%)			0 (0%)	0 (0%)	1.00		0 (0%)	
		no *n* (%)			32 (97%)	66 (98%)	0.56		98 (98%)	
		no answer *n* (%)			1 (3%)	0 (0%)	0.15		1 (1%)	

*n*—number of subjects; * *p* ≤ 0.05—statistically significant value; * other rare syndromes include Angelman syndrome, Arnold Chiari syndrome, fragile X syndrome, congenital malformation syndrome, Prader–Willi syndrome, Silver–Russell syndrome, and metachromatic leukodystrophy. Certain participants in the study group had from 7 to 12 permanent and from 0 to 4 primary anterior teeth (incisors and canines) present in the oral cavity. In total, they had 785 permanent incisors as well as 363 permanent and 27 primary canines. Twenty-two subjects had mixed dentition, while seventy-eight had permanent dentition.

**Table 3:** Number of teeth, in particular groups, affected by traumatic injuries or complications of trauma to primary teeth.

Groups of Teeth		Study Group					Control Group					Total				
		Maxillary (Mx) Teeth		Mandibular (Mb) Teeth		*p* Maxillary vs. Mandibular Teeth	Maxillary (Mx) Teeth		Mandibular (Mb) Teeth		*p* Maxillary vs. Mandibular Teeth	Study Group		Control Group		*p* Study vs. Control Group
		N	%	N	%		N	%	N	%		N	%	N	%	
Incisors	Total number of permanent teeth	389	100.00	396	100.0	-	504	100.0	506	100.0	-	785	100.0	1010	100.0	-
	Teeth with traumatic injuries	17	4.4	0	0.0	<0.001 *	16	3.2	4	0.8	0.02	17	2.2	20	2.0	0.07
	Teeth with opacities	5	1.3	0	0.0	0.046 *	8	1.6	3	0.6	0.36	5	0.6	11	1.1	0.68
	Teeth with hypoplasia	1	0.3	0	0.0	0.37	0	0.0	1	0.2	0.31	1	0.1	1	0.1	0.80
	Total number of primary teeth	0	0.0	0	0.0	1.00	3	100.0	0	0.0	-	0	0.0	3	100.0	
	Teeth with traumatic injuries	0	0.0	0	0.0	1.00	0	0.0	0	0.0	1.00	0	0.0	0	0.0	1.00
Canines	Total number of permanent teeth	181	100.0	182	100.0	-	184	100.0	210	100.0	-	363	100.0	394	100.0	-
	Teeth with traumatic injuries	0	0.0	0	0.0	1.00	0	0.0	0	0.0	1.00	0	0.0	0	0.0	1.00
	Teeth with opacities	0	0.0	0	0.0	1.00	0	0.0	0	0.0	1.00	0	0.0	0	0.0	1.00
	Teeth with hypoplasia	0	0.0	0	0.0	1.00	0	0.0	0	0.0	1.00	0	0.0	0	0.0	1.00
	Total number of primary teeth	16	100.0	11	100.0	-	64	100.0	41	100.0	-	27	100.0	105	100.0	-
	Teeth with traumatic injuries	1	6.2	0	0.0	0.41	0	0.0	0	0.0	1.00	1	3.7	0	0.0	<0.001 *
Total	Total number of permanent teeth	570	100.0	578	100.0	-	688	100.0	716	100.0	-	1148	100.0	1404	100.0	-
	Teeth with traumatic injuries	17	3.0	0	0.0	0.006 *	16	2.3	4	0.6	0.12	17	1.5	20	1.4	0.89
	Teeth with opacities	5	0.9	0	0.0	<0.001 *	8	1.2	3	0.4	0.60	5	0.4	11	0.8	0.28
	Teeth with hypoplasia	1	0.2	0	0.0	0.31	0	0.0	1	0.1	0.40	1	0.1	1	0.1	0.85
	Total number of primary teeth	16	100.0	11	100.0	-	67	100.0	41	100.0	-	27	100.0	108	100.0	-
	Teeth with traumatic injuries	1	6.2	0	0.0	0.41	0	0.0	0	0.0	1.00	1	3.7	0	0.0	<0.001 *

* *p* ≤ 0.05—statistically significant value.

**Table 4:** Number of teeth with traumatic injury in subjects.

Number of Teeth with Traumatic Injury	Number of Patients								
	Study Group			Control Group			Total		
	Females *n* = 33	Males *n* = 67	*p* Females vs. Males	Females *n* = 59	Males *n* = 69	*p* Females vs. Males	Study group *n* = 100	Control Group *n* = 128	*p* Study vs. Control Group
1	3 (9%)	11 (16%)	0.34	2 (3%)	5 (7%)	0.31	14 (14%)	7 (5%)	0.02 *
2	0 (0%)	2 (3%)	0.31	3 (5%)	2 (3%)	0.56	2 (2%)	5 (4%)	0.39
3	0 (0%)	0 (0%)	1.00	1 (2%)	0 (0%)	0.40	0 (0%)	1 (1%)	0.31
Total	3 (9%)	13 (19%)	0.19	6 (10%)	7 (10%)	0.99	16 (16%)	13 (10%)	0.17

*n*—number of subjects; * *p* ≤ 0.05—statistically significant value.

## Data Availability

The original contributions presented in this study are included in the article; further enquiries can be directed to the corresponding authors.

## Abbreviations

The following abbreviations are used in this manuscript:

ADHD	attention deficit hyperactivity disorder
EVA	ethylene-vinyl acetate
FDI	Fédération Dentaire Internationale (World Dental Federation)
MATP	Motor Activity Training Program
MT	teeth missing due to caries
*n*	number of subjects
*p*	statistically significant value
WHO	World Health Organization
